# Author Correction: The distribution and lifetime of powerful radio galaxies as a function of environment and redshift

**DOI:** 10.1038/s41598-019-41871-1

**Published:** 2019-04-16

**Authors:** David Garofalo, Chandra B. Singh, Alexa Zack

**Affiliations:** 1 Department of Physics, Kennesaw State University, Marietta, GA 30060 USA; 2 The Raymond and Beverly Sackler School of Physics and Astronomy, Tel Aviv University, Tel Aviv, 69978 Israel

Correction to: *Scientific Reports* 10.1038/s41598-018-33532-6, published online 10 October 2018

In this Article, there is an error in the legend of Figure 3:

“The two FRII HERGs in blue correspond to Fig. 2 paths a & b higher jet power, smaller lifetime, and path c, lower jet power, longer lifetime.”

should read:

“The two FRII HERGs in blue correspond to Figure 2 path c higher jet power, smaller lifetime, and paths a & b, lower jet power, longer lifetime”

